# Finding a balance in reduced toxicity hematopoietic stem cell transplantation for thalassemia: role of infused CD3+ cell count and immunosuppression

**DOI:** 10.1038/s41409-024-02219-0

**Published:** 2024-02-07

**Authors:** Barbara Meissner, Peter Lang, Peter Bader, Manfred Hoenig, Ingo Müller, Roland Meisel, Johann Greil, Martin G. Sauer, Markus Metzler, Selim Corbacioglu, Birgit Burkhardt, Matthias Wölfl, Brigitte Strahm, Kinan Kafa, Oliver Basu, Holger N. Lode, Bernd Gruhn, Holger Cario, Ann-Kathrin Ozga, Martin Zimmermann, Andrea Jarisch, Rita Beier

**Affiliations:** 1 Department of Pediatric Hematology and Oncology, Hannover Medical School, Hannover, Germany; 2 Department Hematology/Oncology, University Children’s Hospital Tuebingen, Tuebingen, Germany; 3 Department for Children and Adolescents, Division for Stem Cell Transplantation, Immunology and Intensive Care Medicine, Goethe University Frankfurt, University Hospital, Frankfurt, Germany; 4 Department of Pediatrics, University Medical Center Ulm, Ulm, Germany; 5 Division of Pediatric Stem Cell Transplantation and Immunology, University Medical Center Hamburg-Eppendorf, Hamburg, Germany; 6 Devision of Pediatric Stem Cell Therapy, Department of Pediatric Oncology, Hematology and Clinical Immunology, Medical Faculty, Heinrich-Heine-University, Duesseldorf, Germany; 7 University Children’s Hospital Heidelberg, Heidelberg, Germany; 8 Department of Pediatrics and Adolescent Medicine, University Hospital Erlangen, Erlangen, Germany; 9 Department of Pediatric Hematology, Oncology and Stem Cell Transplantation, University of Regensburg, Regensburg, Germany; 10 Pediatric Hematology and Oncology, University Hospital Muenster, Muenster, Germany; 11 University Children’s Hospital Wuerzburg, Wuerzburg, Germany; 12 Department of Pediatrics and Adolescent Medicine, Division of Pediatric Hematology and Oncology, Medical Center, Faculty of Medicine, University of Freiburg, Freiburg, Germany; 13 Pediatric Hematology and Oncology, Martin Luther University Halle-Wittenberg, Halle, Germany; 14 University Children’s Hospital Essen, Essen, Germany; 15 Department of Pediatric Oncology and Hematology, University Medicine Greifswald, Greifswald, Germany; 16 Department of Pediatrics, Jena University Hospital, Jena, Germany; 17 Institute of Medical Biometry and Epidemiology, University Medical Center Hamburg–Eppendorf, Hamburg, Germany

**Keywords:** Anaemia, Stem-cell therapies

## Abstract

We performed a retrospective analysis on 124 patients with transfusion-dependent thalassemia who were registered in the German pediatric registry for stem cell transplantation. All patients underwent first allogeneic hematopoietic stem cell transplantation (HSCT) between 2011 and 2020 and belonged mainly to Pesaro risk class 1–2. Four-year overall (OS) and thalassemia-free survival (TFS) were 94.5% ± 2.9% and 88.0% ± 3.4% after treosulfan-fludarabine-thiotepa- and 96.9% ± 3.1% (*P* = 0.763) and 96.9% ± 3.1% (*P* = 0.155) after busulfan-fludarabine-based conditioning. Mixed chimerism below 75% occurred predominantly in treosulfan-based regimens (27.5% versus 6.2%). OS and TFS did not differ significantly between matched sibling, other matched family and matched unrelated donor (UD) HSCTs (OS: 100.0%, 100.0%, 96.3% ± 3.6%; TFS: 96.5% ± 2.4%, 90.0% ± 9.5%, 88.9% ± 6.0%). However, mismatched UD-HSCTs performed less favorable (OS: 84.7% ± 7.3% (*P* = 0.029); TFS: 79.9% ± 7.4% (*P* = 0.082)). We generated a scoring system reflecting the risk to develop mixed chimerism in our cohort. The main risk-reducing factors were a high CD3+ cell count (≥6 × 10^7^/kg) in the graft, busulfan-conditioning, pre-conditioning therapy and low-targeted ciclosporin A trough levels. Acute GvHD grade III-IV in treosulfan-based concepts predominantly occurred in patients with UD and reduced GvHD prophylaxis but not in the context of high CD3+ cell doses. Taken together, this information might be used to develop more risk-adapted HSCT regimens for thalassemia patients.

## Introduction

Conventional therapy for patients with transfusion dependent thalassemia (TDT) has to be lifelong and often results in iron overload and severe organ dysfunction leading to significant morbidity and mortality over time [1, 2]. Besides promising results in gene therapy in clinical trials [3–6], hematopoietic stem cell transplantation (HSCT) is the only curative treatment option that is widely available and has been performed for decades in TDT [7]. Initially, myeloablative conditioning regimens consisting of busulfan and cyclophosphamide followed by HLA-matched sibling donor (MSD) HSCT represented the standard of care in TDT patients [8–12]. Despite chelation therapy, previous blood transfusions and subsequently organ damage due to iron overload compromise HSCT outcome [10]. Accordingly, signs of inadequate chelation therapy and increased iron overload leading to endorgan damage have been used for risk stratification (Pesaro [13]). High rates of graft failure (GF) and treatment-related mortality (TRM) pose significant challenges in high risk patients [12, 13]. In addition, a healthy MSD is not available for the majority of patients. With progress in high resolution HLA typing and supportive management as well as application of risk-adapted protocols, the outcome after HSCT from a well-matched unrelated donor (UD) has become comparable to results obtained with a MSD although higher rates of complications and graft versus host disease (GvHD) are frequently observed. [7, 14–17] Due to the unfavorable toxicity profile of busulfan (lung, brain, gonadal, sinusoidal obstruction syndrome (SOS)), several transplant centers have started to use reduced-toxicity protocols with treosulfan, fludarabine and thiotepa (TFT) instead. These concepts are characterized by more intensive immunosuppression with lower organ toxicity [18, 19]. On the other hand, there is some concern in terms of increased rates of mixed chimerism (MC) and GF in treosulfan-based concepts in TDT [20]. These issues prompted us to conduct this retrospective analysis of HSCT in thalassemia patients reported to the German pediatric registry for stem cell transplantation and cell therapy (PRSZT) between 2011 and 2020.

First, we assessed newer concepts of matched (10/10) unrelated donor (MUD) and mismatched (9/10) unrelated donor (MMUD) HSCT in comparison with standard MSD-HSCT regarding outcome (overall survival (OS), thalassemia-free survival (TFS), thalassemia-free and persisting chronic-GvHD-free survival (TGFS), MC, GF, TRM and GvHD). Second, we compared treosulfan- and busulfan-based conditioning regimens in order to identify critical differences that need to be addressed by the concept. Finally, we wanted to identify factors preventing MC and GF without leading to severe GvHD (especially in TFT-based regimens) to support the development of improved, risk-adapted concepts of low toxicity.

## Methods

### Data source

This is a retrospective multicenter registry analysis from the German PRSZT, which is a nationwide association of pediatric transplant centers. Informed consent for registration and data collection was obtained from all patients and/or their legal guardians following the principles of the Declaration of Helsinki (IRB approval #1979–2013).

### Patients

Patients who received a first allogeneic HSCT for TDT between June 2011 and February 2020 were included. HSCTs from MSD, matched (10/10) family donor other than siblings (MFD), MUD or 9/10 MMUD were analyzed only. A follow up for at least 12 months was aimed for in all survivors.

### Outcomes and definitions

The primary endpoints were OS (time from HSCT to death of any cause/last follow up), TFS (time from HSCT to graft failure (recurrence of transfusion dependency) or death – whichever occurred first - or last follow-up), as well as TGFS (definition as TFS but occurrence of persisting chronic GvHD (cGvHD) as additional event). Secondary endpoints included GF, lowest and last chimerism, incidence of acute GvHD grade III-IV (aGvHD III-IV), extensive chronic GvHD (extCGvHD), and cytomegalovirus-reactivations. Lowest chimerism was defined as lowest percentage of donor derived hematopoiesis ever documented in the post-transplant period and MC as <95% of donor cells. aGvHD and cGvHD severity were graded according to Glucksberg criteria [21]. Patients were stratified into Pesaro risk classification [13] except patients for whom relevant data were missing. Grafalon® (formerly ATG-Fresenius, ATG-F) 60 mg/kg body weight (b.w.) and Thymoglobuline® 10 mg/kg b.w. were considered as high dose, everything below as low dose ATG. Based on the initially aimed, mean ciclosporin A (CSA) trough target level we divided the patients into three groups (≤100 µg/l (low), 101–149 µg/l (medium) and ≥150 µg/l (high)).

### Statistical methods

Demographic, baseline and treatment variables as well as outcome parameters were reported for the entire study population and in the following separately according to donor type and conditioning regimen. Categorical data were summarized by absolute and relative frequencies and compared by Fisher’s exact (chi square) test. For continuous variables, median (range) was calculated and then compared using Mann–Whitney-*U* or Kruskal–Wallis *H* Test. Survival probabilities were estimated by Kaplan–Meier methodology and compared using the log-rank test. The impact of the following variables on outcome were assessed: recipient age and sex, last serum ferritin level before HSCT, liver iron concentration [22, 23], Pesaro risk classification, sex- as well as cytomegalo- and Eppstein-Barr virus status-matching between recipient and donor, donor type, pre-conditioning therapy, conditioning regimen, antithymocyte globulin (ATG) application, dosage of ATG application, stem cell source and cell counts, mean targeted CSA trough level, and GvHD prophylaxis. Multivariable Cox regression analysis was limited to cytomegalovirus reactivation due to the small number of events regarding OS, TFS, aGvHD III-IV, extCGvHD and GF with several subgroups having no events. Regarding mixed chimerism a multivariable logistic regression analysis was performed which included all variables that were significant (*p* < 0.05) in univariable analysis. Median follow-up was calculated using the inverse Kaplan–Meier method. *P*-values were 2-sided and were considered statistically significant if <0.05. Statistical analyses were performed with SPSS version 27.0 (IBM SPSS Inc, IL, USA), SAS version 9.4 (Cary, NC, USA) and R version 4.1.2.

## Results

### Patient and donor characteristics

124 pediatric and young adult patients received allogeneic HSCT for TDT between June 2011 and February 2020 at 15 different pediatric transplant centers. Characteristics for the whole group as well as stratified according to donor subgroups are presented in Table 1 and Supplementary Table 1.

**Table 1:** Patient and HSCT characteristics as well as outcome in the entire cohort of 124 patients and differences according to donor subgroup.

	*n* (%) Total *N* = 124	*n* (%) MSD *N* = 57	*n* (%) MFD *N* = 10	*n* (%) MUD *N* = 27	*n* (%) MMUD *N* = 30	Descriptive *P*
Patient age at HSCT, years, median (range)	8.4 (1.4–28.1)	9.2 (1.5–28.1)	6.6 (1.4–16.5)	7.6 (1.7–23.7)	9.6 (2.0–18.1)	0.649
Patient sex, female, *n* (%)	56 (45.2)	23 (40.4)	5 (50.0)	11 (40.7)	17 (56.7)	0.486
Serum ferritin ≥ 3000 µg/l, *n* (%)	25 (20.3)	10 (17.5)	2 (20.0)	3 (11.1)	10 (34.5)	0.169
Pesaro class, *n* (%)						
1	4 (3.4)	3 (5.4)	0 (0.0)	1 (3.8)	0 (0.0)	0.099
1 or 2	64 (53.8)	22 (39.3)	6 (60.0)	16 (61.6)	20 (74.1)	
2	25 (21.0)	18 (32.1)	2 (20.0)	4 (15.4)	1 (3.7)	
2 or 3	11 (9.2)	6 (10.7)	1 (10.0)	3 (11.5)	1 (3.7)	
3	0 (0.0)	0.0 (0.0)	0 (0.0)	0 (0.0)	0 (0.0)	
≥ 16 years	15 (12.6)	7 (12.5)	1 (10.0)	2 (7.7)	5 (18.5)	
Not evaluable	5	1	0	1	3	
Stem cell source, n (%)						
BM only	94 (75.8)	50 (87.7)	9 (90.0)	20 (74.1)	15 (50.0)	<0.001
Cord blood +/− BM	4 (3.2)	4 (7.0)	0 (0.0)	0 (0.0)	0 (0.0)	
PBSC without TCD	15 (12.1)	3 (5.3)	0 (0.0)	4 (14.8)	8 (26.7)	
PBSC with TCD	11 (8.9)	0 (0.0)	1 (10.0)	3 (11.1)	7 (23.3)	
CD 3 + , x10^7^/kg, median (range)						
BM only	4.5 (1.3–30.4)	4.1 (1.3–12.5)	5.1 (1.4–7.4)	4.5 (2.6–30.4)	4.8 (2.1–13.3)	<0.001
PBSC with TCD	5.0 (0.1–18.2)	-	18.2	5.0 (0.5–5.1)	2.7 (0.1–5.5)	
PBSC without TCD	27.0 (15.0–52.5)	15.0	-	41.8 (20.0–52.5)	26.8 (17.6–50.6)	
All except CB	4.7 (0.1–52.5)	4.2 (1.3–15.0)	5.1 (1.4–18.2)	5.0 (0.5–52.5)	5.0 (0.1–50.6)	0.062
Pre-conditioning, *n* (%)						
Yes	53 (42.7)	23 (40.4)	3 (30.0)	12 (44.4)	15 (50.0)	0.711
No	71 (57.3)	34 (59.6)	7 (70.0)	15 (55.6)	15 (50.0)	
Conditioning, *n* (%)						
BF-based	32 (25.8)	17 (29.8)	3 (30.0)	5 (18.5)	7 (23.3)	0.701
TFT	92 (74.2)	40 (70.2)	7 (70.0)	22 (81.5)	23 (67.7)	
GvHD prophylaxis, *n* (%)						
CSA only	3 (2.4)	3 (5.3)	0 (0.0)	0 (0.0)	0 (0.0)	0.351
CSA + normal MTX	65 (52.3)	28 (49.1)	5 (50.0)	18 (66.7)	14 (46.7)	
CSA + red MTX +/-MMF	9 (7.3)	3 (5.3)	1 (10.0)	2 (7.4)	3 (10.0)	
CSA + MTX (amount unclear)	1 (0.8)	0 (0.0)	0 (0.0)	0 (0.0)	1 (3.3)	
CNI + MMF	22 (17.7)	15 (26.3)	1 (10.0)	3 (11.1)	3 (10.0)	
Three agents	24 (19.4)	8 (14.0)	3 (30.0)	4 (14.8)	9 (30.0)	
Mean targeted trough level of CSA, µg/l, *n* (%)						
≤100	14 (11.6)	8 (14.8)	0 (0.0)	4 (14.8)	2 (6.7)	0.705
101–149	73 (60.3)	34 (63.0)	6 (60.0)	15 (55.6)	18 (60.0)	
≥150	34 (28.1)	12 (22.2)	4 (40.0)	8 (29.6)	10 (33.3)	
Graft failure, *n* (4y-probability, %)	8 (6.6)	2 (3.5)	1 (10.0)	2 (7.4)	3 (10.8)	0.578
Lowest chimerism, *n* (%)						
95–100%	65 (52.8)	17 (29.8)	5 (50.0)	20 (74.1)	23 (79.3)	<0.001
75–94%	31 (25.2)	27 (47.4)	0 (0.0)	3 (11.1)	1 (3.4)	
50–74%	6 (4.9)	3 (5.3)	1 (10.0)	1 (3.7)	1 (3.4)	
<50%	21 (17.1)	10 (17.5)	4 (40.0)	3 (11.1)	4 (13.8)	
Not evaluable	1	0	0	0	1	
Last chimerism, *n* (%)						
95–100%	91 (74.0)	38 (66.7)	6 (60.0)	22 (81.5)	25 (86.2)	0.028
75–94%	13 (10.6)	11 (19.3)	0 (0.0)	2 (7.4)	0 (0.0)	
50–74%	4 (3.3)	2 (3.5)	2 (20.0)	0 (0.0)	0 (0.0)	
<50%	15 (12.1)	6 (10.5)	2 (20.0)	3 (11.1)	4 (13.8)	
Not evaluable	1	0	0	0	1	
Cell therapy, *n* (%)						
None	103 (83.7)	45 (78.9)	8 (80.0)	23 (85.2)	27 (93.1)	0.204
Autologous rescue	1 (0.8)	0 (0.0)	0 (0.0)	0 (0.0)	1 (3.4)	
Only DLI	10 (8.1)	8 (14.0)	1 (10.0)	1 (3.7)	0 (0.0)	
Allogeneic boost +/− DLI	9 (7.4)	4 (7.0)	1 (10.0)	3 (11.1)	1 (3.4)	
Not evaluable	1	0	0	0	1	
aGvHD, *n* (1y-probability, %)						
II-IV	20 (16.3)	3 (5.3)	1 (10.0)	6 (22.2)	10 (34.5)	0.002
III-IV	13 (10.6)	0 (0.0)	1 (10.0)	6 (22.2)	6 (20.7)	0.003
extCGvHD, *n* (4y-probability, %)	11 (9.9)	2 (5.0)	1 (10.0)	5 (19.2)	3 (11.1)	0.153
Complications^a^, *n* (%)	45 (36.3)	14 (24.6)	4 (40.0)	12 (44.4)	15 (50.0)	0.079

*ATG* antithymocyte globulin, *aGvHD* acute graft versus host disease, *ANC* absolute neutrophil count, *BF-based* busulfan-fludarabine-based, *BM* bone marrow, *CB* cord blood, *CNI* Calcineurin Inhibitor, *CMV* cytomegalovirus, *CSA* ciclosporin A, *D* donor, *DLI* donor lymphocyte infusion, *EBV* Epstein Barr virus, *extCGvHD* extended chronic graft versus host disease, *GvHD* graft versus host disease, *HSCT* hematopoietic stem cell transplantation, *LIC* liver iron concentration, *MFD* matched family donor other than sibling, *MMF* mycophenolate mofetil, *MSD* matched sibling donor, *MUD* matched unrelated donor 10/10, *MMUD* mismatched unrelated donor 9/10, *PBSC* peripheral blood stem cells, *R* recipient, *red MTX* reduced methotrexate, *SOS* hepatic sinusoidal obstruction syndrome, *TCD* ex vivo T-cell depletion, *TFS* thalassemia-free survival, *TFT* treosulfan, fludarabine and thiotepa, *TGFS* thalassemia-free survival without persisting chronic GvHD at last follow up, *TRM* treatment related mortality.

^a^including aGvHD III-IV, cGvHD, mechanical ventilation, oxygen therapy, inotropic support, dialysis, SOS, encephalopathy, ileus, gastrointestinal bleeding.

### Transplant characteristics

Details regarding HSCT procedure are also shown in Table 1. 57 patients (46%) received their graft from an UD. Conditioning regimens including busulfan was applied in 32 patients and always contained a busulfan-fludarabine (BF)-based approach (details see Supplementary results). All 92 patients with treosulfan-based conditioning received TFT. 109 of 119 patients with ATG serotherapy received the last dose on day-3 or later.

### Overall survival, TFS and TGFS

**Fig. 1:**
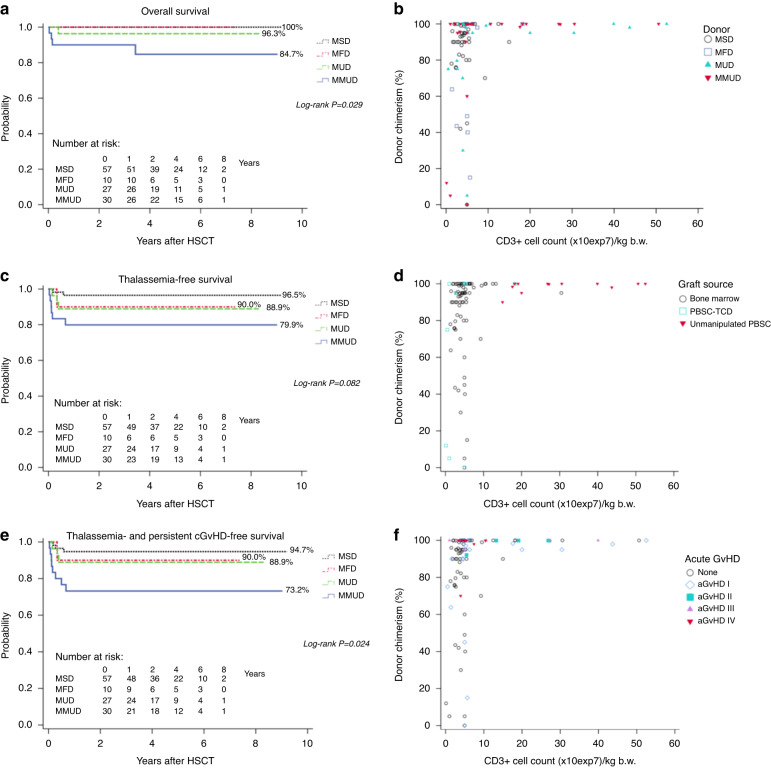
Outcome according to donor and lowest chimerism in relation to CD3+ cell count in the entire cohort. **a**–**c** Presentation of OS, TFS and TGFS according to donor groups. (**a)** OS (**b**) TFS and (**c**) TGFS. MFD matched family donor other than sibling, MSD matched sibling donor, MUD matched unrelated donor 10/10, MMUD mismatched unrelated donor 9/10. **d**–**f** Relationship of lowest donor chimerism (percentage of donor cells) and CD3+ cell count in the graft. In 99 patients with available CD3+ cell count, the relationship of lowest donor chimerism and CD3+ cell count in the graft (x10^7^/kg body weight) is depicted with the specification of (**d**) donor (**e**) graft source (**f**) acute GvHD. MFD matched family donor other than sibling, MSD matched sibling donor, MUD matched unrelated donor 10/10, MMUD mismatched unrelated donor 9/10, PBSC peripheral blood stem cells, TCD ex vivo T-cell depletion.

The outcome stratified by donor type is shown in Fig. 1a–c. Median follow up of surviving patients was 3.2 years (range 0.6–9.2). 4y-OS in the entire cohort was excellent (95.1%) with four deaths occurring after 1st and one after 2nd HSCT. Death in two patients were associated with aGvHD grade IV. The other two died due to systemic candida infection and systemic cytomegalovirus disease with pulmonary failure, respectively. All four patients who died after 1st HSCT were older than 12 years and had received highly immunosuppressive regimens with pre-conditioning and either high-dose ATG or post-transplant cyclophosphamide with low-dose ATG serotherapy. There was a trend towards worse TFS with increasing HLA disparity (Fig. 1b). When analyzing TGFS, this trend became significant with patients following MMUD transplant reaching only 73.2% (Fig. 1c).

### Engraftment

The overall neutrophil engraftment rate was 99.2%. One patient died on day +13 before reaching neutrophil engraftment. Time to neutrophil-engraftment was significantly delayed after MSD-HSCTs (Supplementary Table 1) as well as when cord blood (CB) or bone marrow (BM) grafts were used (Supplementary Fig. 1a). Median time to platelet engraftment was also mainly dependent on stem cell source: patients receiving peripheral blood stem cells (PBSC, *n* = 26) had fast median platelet engraftment of 19 days, whereas this was significantly delayed (median 29 days, *P* = 0.011) in patients with BM (*n* = 94, see also Supplementary Fig. 1b).

### Graft failure and chimerism

Eight patients suffered from GF (Table 1). One patient following MMUD transplant achieved only neutrophil engraftment 2 weeks after HSCT, but subsequently rejected the graft a few days later. The remaining (7/8) showed sustained graft function during the first months after HSCT, even with complete donor chimerism in the majority of these patients. However, this was followed by autologous recovery and secondary GF between 6 weeks and 9 months after HSCT requiring regular red cell transfusions. All GF (5 after BM and 3 after ex vivo T-cell depleted PBSC (PBSC-TCD)) occurred in the group of 35 patients that had received TFT conditioning without pre-conditioning therapy and less than 6 × 10^7^/kg b.w. CD3+ cells in the graft as well as medium to high targeted CSA trough levels (Supplementary Tables 2, 3). The apparent association of GF and TFS with sex was caused by more transplantations of girls in regimens that were of higher risk.

Mixed donor chimerism was a frequently observed phenomenon (Tables 1, 2; Fig. 1d–f; Supplementary Table 4; Supplementary Fig. 2), especially in MSD-HSCT (with TFT-conditioning (27/40)). However, risk of secondary graft failure in pre-existing mixed chimerism increased with HLA-disparity (MSD 5% (2/40), MFD 20% (1/5), MUD 29% (2/7), MMUD 40% (2/5)). Risk factors for the development of mixed chimerism (<95%) included MSD, missing pre-conditioning, low CD3+ cell count, and higher CSA levels (Table 3). Risk of mixed chimerism below 75% was higher with TFT-conditioning.

**Table 2:** Differences in patient and HSCT characteristics as well as outcome in 124 patients receiving different conditioning regimens.

	*n* (%) TFT *N* = 92	*n* (%) BF-based *N* = 32	Descriptive *P*
Patient age at HSCT, years, median (range)	8.4 (1.4–28.1)	9.7 (1.5–18.7)	0.837
Patient sex, female, *n* (%)	43 (46.7)	13 (40.6)	0.681
Serum ferritin ≥3000 µg/l, *n* (%)	14 (15.4)	11 (34.4)	0.039
Pesaro class, *n* (%)			
1	3 (3.3)	1 (3.4)	0.528
1 or 2	52 (57.8)	12 (41.4)	
2	17 (18.9)	8 (27.6)	
2 or 3	8 (8.9)	3 (10.3)	
3	0 (0.0)	0 (0.0)	
≥ 16 years	10 (11.1)	5 (17.2)	
Not evaluable	2	3	
Donor			
MSD	40 (43.5)	17 (53.1)	0.701
MFD	7 (7.6)	3 (9.4)	
MUD 10/10	22 (23.9)	5 (15.6)	
MMUD 9/10	23 (25.0)	7 (21.9)	
Stem cell source, n (%)			
BM only	68 (73.9)	26 (81.3)	0.758
Cord blood +/− BM	3 (3.3)	1 (3.1)	
PBSC without TCD	13 (14.1)	2 (6.3)	
PBSC with TCD	8 (8.7)	3 (9.4)	
CD 3+, x10^7^/kg, median (range)			
BM only	4.5 (1.4–30.1)	4.4 (1.3–12.5)	<0.001
PBSC with TCD	5.0 (0.1–5.5)	2.7 (0.5–18.2)	
PBSC without TCD	28.7 (15.0–52.5)	22.4 (17.6–27.2)	
All except CB	5.0 (0.1–52.5)	4.4 (0.5–27.2)	0.236
Pre-conditioning			
Yes	34 (37.0)	19 (59.4)	0.038
No	58 (63.0)	13 (40.6)	
GvHD prophylaxis, *n* (%)			
CSA only	3 (3.3)	0 (0.0)	<0.001
CSA + normal MTX	63 (68.5)	2 (6.2)	
CSA + red MTX + /-MMF	9 (9.8)	0 (0.0)	
CSA + MTX (dosage unclear)	0 (0.0)	1 (3.1)	
CNI + MMF	12 (13.0)	10 (31.3)	
Three agents	5 (5.4)	19 (59.4)	
Mean targeted trough level of CSA, µg/l, n (%)			
≤100 (low)	14 (15.7)	0 (0.0)	<0.001
101–149 (medium)	63 (70.8)	10 (31.2)	
≥150 (high)	12 (13.5)	22 (68.8)	
Graft failure, *n* (4y-probability, %)	8 (8.9)	0 (0.0)	0.090
Lowest chimerism, *n* (%)			
95–100%	50 (54.9)	15 (46.9)	0.003
75–94%	16 (17.6)	15 (46.9)	
50–74%	5 (5.5)	1 (3.1)	
<50%	20 (22.0)	1 (3.1)	
Not evaluable	1	0	
Last chimerism, *n* (%)			
95–100%	62 (68.1)	29 (90.6)	0.023
75–94%	10 (11.0)	3 (9.4)	
50–74%	4 (4.4)	0 (0.0)	
<50%	15 (16.5)	0 (0.0)	
Not evaluable	1	0	
Cell therapy, *n* (%)			
None	76 (83.5)	27 (84.4)	0.938
Autologous rescue	1 (1.1)	0 (0.0)	
Only DLI	7 (7.7)	3 (9.4)	
Allogeneic Boost +/− DLI	7 (7.7)	2 (6.3)	
Not evaluable	1	0	
aGvHD, *n* (1y-probability, %)			
II-IV	16 (17.6)	4 (12.6)	0.448
III-IV	10 (11.0)	3 (9.4)	0.750
extCGvHD, *n* (4y-probability, %)	7 (8.9)	4 (12.9)	0.380
Complications^a^, *n* (%)	27 (29.3)	18 (56.3)	0.010
4-year overall survival, %	94.5	96.9	0.763
4-year TFS, %	88.0	96.9	0.155
4-year TGFS, %	85.9	93.8	0.244

*ATG* antithymocyte globulin, *aGvHD* acute graft versus host disease, *BF-based* busulfan-fludarabine-based, *BM* bone marrow, *CB* cord blood, *CNI* Calcineurin Inhibitor, *CSA* ciclosporin A, *D* donor, *DLI* donor lymphocyte infusion, *extCGvHD* extended chronic graft versus host disease, *GvHD* graft versus host disease, *HSCT* hematopoietic stem cell transplantation, *MFD* matched family donor other than sibling, *MMF* mycophenolate mofetil, *MSD* matched sibling donor, *MUD* matched unrelated donor 10/10, *MMUD* mismatched unrelated donor 9/10, *PBSC* peripheral blood stem cells, *R* recipient, *red MTX* reduced methotrexate, *TCD* ex vivo T-cell depletion, *TFS* thalassemia-free survival, *TFT* treosulfan, fludarabine and thiotepa, *TGFS* thalassemia-free survival without persisting chronic GvHD at last follow up.

^a^including aGvHD III-IV, cGvHD, mechanical ventilation, oxygen therapy, inotropic support, dialysis, SOS, encephalopathy, ileus, gastrointestinal bleeding.

**Table 3:** Risk factors for mixed donor chimerism in 98 patients with available data analyzed by multivariable logistic regression analysis.

Outcome	Events/evaluable	Odds ratio (95% CI)	*P*
**Mixed donor chimersim < 95%**			
Donor			
Other than MSD	16/59	1.0	0.003
MSD	24/39	4.9 (1.7–14.4)	
Pre-conditioning			
Yes	12/43	1.0	0.006
No	28/55	4.7 (1.6–14.2)	
CD3+ cells in the graft			
≥ 6 × 10^7^	2/26	1.0	0.001
< 6 x10^7^	38/72	15.3 (3.0–79.4)	
Mean targeted trough level of CSA			
≤ 100 µg/l	4/14	1.0	0.005
> 100 µg/l	36/84	9.4 (2.0–43.9)	
**Mixed donor chimerism < 75%**			
Conditioning			
BF-based	2/31	1.0	0.040
TFT	14/67	5.2 (1.1–25.0)	
CD3+ cells in the graft			
≥6 × 10^7^	1/26	1.0	0.041
<6 × 10^7^	15/72	8.9 (1.1–72.4)	

Only variables with *P* < 0.05 are depicted.

*BF-based* busulfan-fludarabine-based, *CI* confidence interval, *CSA* ciclosporin A, *MSD* matched sibling donor, *TFT* treosulfan, fludarabine and thiotepa.

In 56 HSCTs from UD, that were evaluable regarding chimerism, none of 19 patients who received either unmanipulated PBSC (*n* = 12; CD3 + ≥ 6  × 10^7^/kg) or BM with high amounts of CD3+ cells in the transplant (*n* = 7; ≥6  × 10^7^/kg) developed MC. In contrast, in 4/10 HSCT with PBSC-TCD as well as in 9/27 with BM (lower amounts of CD3+ cells in the graft (<6 × 10^7^/kg)) MC was observed.

Next, we analyzed a cohort of 20 patients who received TFT conditioning and HSCT from MSD with ATG, with BM as graft and without pre-conditioning therapy (and therefore represented a homogeneous cohort potentially at higher risk for MC). Seven of eight patients with low targeted CSA levels and early cessation of immunosuppression, had complete chimerism at last follow up and lowest chimerism of at least 93%. None received donor lymphocyte infusions (DLI)/boost. In the other thirteen patients with medium to high targeted CSA levels 6 patients received DLI, only three patients had full chimerism at last follow up (one with DLI/Boost), two patients had GF and 8 patients had last chimerism of 15–90%.

Based on cumulative incidences for GF (Supplementary Tables 2, 3), multivariable logistic regression analysis (Table 3) and on pathophysiological considerations we developed a score potentially suitable to predict the risk of mixed donor chimerism in our patient cohort. The four adjustable variables “myeloablation”, “pre-conditioning”, “CD3+ cell count with graft source” and “mean trough level of targeted CSA” were included (Table 4).

**Table 4:** Lowest donor chimerism and graft failure in relation to scoring system that reflects myeloablation, pre-conditioning therapy, CD3+ cell count with graft source and mean targeted trough level of CSA.

Score	No. patients	% of patients of that score with lowest chimerism of				No. patients with GF
		≥95%	75–94%	50–74%	<50%	
1	2	100	0	0	0	0
2	11	91	0	9	0	0
3	4	75	0	25	0	0
4	38	69	18	8	5	0
5	27	52	40	4	4	0
6	24	21	50	0	29	3
7	16	31	6	0	63	4
8	1	0	0	0	100	1
Evaluable	123					8

Scoring system: myeloablation: busulfan = 0, treosulfan 42 g/m^2^ = 1, treosulfan 36 g/m^2^ = 2; pre-conditioning: azathioprine and hydroxyurea or dexamethasone and fludarabine = 0; azathioprine or hydroxyurea = 1, none = 2; CD3+ cell count with graft source: ≥6 × 10^7^/kg = 0, <6 × 10^7^/kg and unmanipulated PBSC = 1, <6 × 10^7^/kg and BM/CB/PBSC-TCD = 2; mean trough level of targeted CSA: ≤100 µg/l = 0, 101–149 µg/l = 2, ≥150 µg/l = 3.

*GF* graft failure.

### GvHD

In general, severe aGvHD III-IV started early between day +10 and day +40 approximately around the time of neutrophil engraftment. None of the patients with MSD suffered from aGvHD III-IV ((*P* log-rank 0.003) Supplementary Table 2; Table 1). aGvHD III-IV was mainly observed in patients transplanted from UDs (12 of 13). Next to a higher degree of HLA-disparity, also graft source and cell count were significantly different in UDs in comparison to MSDs. PBSC were administered more often and larger amounts of MNC and CD3+ cells were given, in contrast to BM as graft source (Table 1 and Supplementary Table 1). Nevertheless, reduced GvHD prophylaxis (low level of targeted CSA (*P* log-rank 0.007) and/or reduction/replacement of MTX by MMF (*P* log-rank <0.001)) seemed to be the main factor for severe aGvHD III-IV in HSCTs from UD following TFT conditioning (Table 5). Stem cell source and CD3+ cell count in the graft were no major contributors for aGvHD III-IV as only one patient with unmanipulated PBSC and high CD3+ cell count in the graft developed severe aGvHD (Fig. 1e, f) whereas five patients after BM and three after PBSC-TCD transplants. GvHD prophylaxis in patients with UD, TFT concept and unmanipulated PBSC comprised ATG Grafalon® (30–60 mg/kg and last dose mostly given on day-1), CSA with targeted trough level of at least 120 µg/l, and usually three doses of MTX.

**Table 5:** Univariable analysis of acute GvHD III-IV analyzed in patients with unrelated donor and TFT conditioning (*N* = 45).

Outcome Acute GvHD III-IV	1y-Probability aGvHD III-IV,% (95% CI)	Descriptive *P* Log-rank
Sex		
Male	9.5 (2.5–33.0)	0.076
Female	30.4 (15.8–53.4)	
GvHD prophylaxis		
CSA only	NA	<0.001
CSA + normal MTX	6.5 (1.7–23.4)	
CSA + red MTX ( + /−MMF)	60.0 (24.7–94.8)	
CSA + MTX (amount unclear)	0.0 (NA)	
CNI + MMF	60.0 (24.7–94.8)	
Three agents (CSA, MMF, red. MTX)	33.3 (5.5–94.6)	
Mean targeted trough level of CSA (µg/l)		
≤100	66.7 (32.4–95.4)	0.007
101–149	17.2 (7.6–36.6)	
≥150	0.0 (NA)	

Only variables with a *P* < 0.1 are depicted.

*aGvHD* acute graft versus host disease, *CI* confidence interval, *CNI* Calcineurin Inhibitor, *CSA* ciclosporin A, *MMF* mycophenolate mofetil, *NA* not applicable, *red MTX* reduced methotrexate, *TFT* treosulfan, fludarabine and thiotepa.

Chronic GvHD (cGvHD) with systemic treatment (twelve extensive, one limited) also mainly occurred in UDs (Table 1). Extensive cGvHD was often associated with high ferritin of ≥3000 µg/l (*P* log-rank 0.022) before HSCT and the occurrence of severe aGvHD III-IV (*P* log-rank 0.001) (Supplementary Table 2). In 9/12 patients extensive cGvHD resolved.

### Complications

The rate of relevant complications increased in parallel with the gradually increasing HLA disparity between different donor groups (Table 1). In a multivariable analysis of 89 patients at higher risk for cytomegalovirus reactivations (recipient cytomegalovirus IgG positive), patients with MMUD (HR 5.02; 95% CI 1.79–14.07; *P* = 0.002), high dosage of ATG (HR 3.68; 95% CI: 1.22–11.10; *P* = 0.021) or pre-conditioning therapy (HR 3.34; 95% CI: 1.43–7.80; *P* = 0.005) showed a significantly increased risk of cytomegalovirus reactivation (Supplementary Table 5).

### Comparison of BF-based and TFT conditioning concepts

Outcome (OS, TFS, TGFS) was similar in patients with TFT and BF-based conditioning (Table 2), although there was a trend towards more GF, lower TFS and higher rates of MC (27.5% versus 6.2% for lowest chimerism<75%) in TFT-based concepts (Supplementary Fig. 2a). On the other hand, patients with BF-based concepts suffered from almost twice as many severe complications and showed delayed platelet engraftment (Supplementary Table 6). The incidence of severe aGvHD was similar in both conditioning groups but patients with BF-based regimens received more intense GvHD prophylaxis (ATG dose higher, often a third agent in addition to CSA and methotrexate, higher CSA target levels).

## Discussion

Many important insights have been gained from previous large retrospective, multicenter reports in TDT [8–10, 20, 24, 25]. The present study contributes new aspects in particular regarding the interdependence of pre-conditioning therapy, conditioning regimen, stem cell source with CD3+ cell counts, donor type and GvHD prophylaxis. This was made possible by a very granular set of data including, for example, ferritin, CD3+ cell count in the graft, pre-conditioning therapy, details of GVHD prophylaxis, time course of chimerism, post-transplant cellular therapy, cytomegalovirus reactivations and relevant complications. In comparison to other reports [8, 10, 16, 26], the majority of patients had access to well managed conservative treatment before HSCT (Pesaro class 1 or 2, median ferritin 1800 µg/l). With a 4y-OS of 95.1%, 4y-TFS of 90.3% and 4y-TGFS of 87.9% the participating centers achieved a very good overall outcome, especially considering the high proportion of UD (MUD 22%, MMUD 24%). Although outcome with MMUD was significantly inferior supporting other reports [20, 24, 27], MMUD-HSCT seems justifiable if an appropriate conditioning concept is used, even though the combined risk of GvHD and infections is likely to be higher. In addition, MC occurred frequently (47.2%) leading occasionally to the administration of post-transplant cellular therapy (19/124). This prompted us to focus on preventive and predisposing settings, which eventually led us to develop an algorithm in order to assist in the development of future risk-adopted immunosuppressive strategies.

When comparing BF- and TFT-based therapies in our cohort, both approaches achieved similar results. However, the two regimens had different challenges (TFT: more MC and GF; BF-based: more complications and need for more intense GvHD prophylaxis [28, 29]). These issues underline that patient outcome largely depended on the adaption of GvHD prophylaxis to the conditioning concept, timely management of mixed chimerism and on the handling of potential complications.

Primary graft failures and rejections were no major problem in our cohort, which was dominated by highly immunosuppressive conditioning regimens. On the other hand, mixed chimerism was a significant concern, especially in MSD-HSCT with TFT conditioning. In addition to less differences in minor histocompatibility antigens in MSD and therefore reduced T cell alloreactivity, the pediatric setting with mainly use of bone marrow in MSD-HSCT leading to low CD3+ and mononuclear cell count in the graft probably also had an impact on high rates of MC and delayed neutrophil engraftment in this subgroup.

In order to better understand the interaction of influencing factors on donor chimerism, we performed a multivariable regression analysis regarding mixed chimerism. Also, we propose a scoring system that assess risk for MC in our cohort. It is based on four major influencing variables: high CD3+ cell count in the graft (≥6 × 10^7^/kg), myeloablation with busulfan, pre-conditioning therapy as well as low-targeted CSA levels had protective impact. Unmanipulated PBSC, myeloablation with busulfan, and pre-conditioning therapy are general risk reducing factors for MC/GF that already have been described in other reports [11, 20, 26]. However, the T cell count in the graft seemed to be an important protective parameter for MC in our cohort. So far, only one report has described the association of high CD3+ cell count in the graft and reduced GF rate in TDT [30]. This was a single center study that differed significantly from ours in many aspects. The protective effect of low-targeted CSA level in our cohort is mainly attributed to TFT concepts. If these regimens are combined with reduced CD3+ cell count and are given without pre-conditioning therapy a well-adapted concept of GvHD prophylaxis seems to be of major importance.

In MSD-HSCT with Pesaro risk class 1–2 and well-controlled iron load before HSCT, a regimen with TFT, ATG, BM and without pre-conditioning therapy might be feasible, because CSA levels can be kept low and rapidly reduced due to the very low risk of severe GvHD. In UD-HSCT, however, the risk of severe aGvHD III-IV is significantly increased. Even with bone marrow as graft source, higher targeted CSA levels seem to be necessary especially at the time of engraftment and shortly thereafter. As a consequence, other measures such as either application of pre-conditioning or higher CD3+ cell count in the graft (for instance by application of unmanipulated PBSC [15, 26]) might be necessary to avoid MC and secondary graft failure. Unmanipulated PBSC seem promising in combination with treosulfan-based regimens [26] and high resolution HLA typing because risk of infections is not elevated and risk of severe aGvHD or cGvHD can be balanced by adequate GvHD prophylaxis.

The combination of extensive pre-conditioning therapy and high amount of ATG although protective against GF might be challenging due to higher incidences of cytomegalovirus reactivations and possibly also other infectious complications as well as TRM. Based on our retrospective data, such concepts might be considered primarily for high risk patients regarding GF (such as MMUD and/or Pesaro class 3) with careful virus monitoring and antiinfective prophylaxis.

This retrospective, multicenter analysis is limited by the comparison of various concepts differing in several HSCT characteristics. In addition, the explanatory power of the statistical analysis was largely limited due to small number of events. Nevertheless, we have performed a very detailed data analysis which enabled us to describe important protective factors for secondary GF and MC such as CD3+ cell count in the graft or GvHD prophylaxis. These aspects may be used for optimization of risk-adapted conditioning regimens and the development of randomized studies in TDT. Ultimately, clinical experience with specific concepts, such as management of concept-specific complications and control of GvHD prophylaxis over time, certainly plays an essential role in final outcome.

### Supplementary information

Finding a balance in reduced toxicity hematopoietic stem cell transplantation for thalassemia: role of infused CD3+ cell count and immunosuppression

## Data Availability

The patient-level data used for this study are not publicly available due to privacy restrictions. The aggregated data generated during the current study are available from the corresponding author on reasonable request.
