# Supplementary material for: Finding a balance in reduced toxicity hematopoietic stem cell transplantation for thalassemia: role of infused CD3+ cell count and immunosuppression
Source: Bone Marrow Transplant. 2024 Feb 7;59(5):587–96. doi: 10.1038/s41409-024-02219-0 (PMC11073967; doi:10.1038/s41409-024-02219-0)
Supplement: Supplementary file 1 — Finding a balance in reduced toxicity hematopoietic stem cell transplantation for thalassemia: role of infused CD3+ cell count and immunosuppression [file 41409_2024_2219_MOESM1_ESM.pdf]

SUPPLEMENTARY DATA

SUPPLEMENTARY METHODS

Definitions

Time to cytomegalovirus reactivation was time from date of HSCT to date of reactivation or last follow up. Time to neutrophil engraftment was defined as the time between HSCT and the first of 3 consecutive days achieving a neutrophil count of > 500/µl, time to platelet engraftment as first of three consecutive days with a platelet count of >20/nl unsupported for 7 days. The cut point of 6 x 10^7/kg CD3+ cells was determined by the probability of predicting full chimerism (Fig. 1d-f and Supplementary Fig. 2). Pesaro classification was performed as described in the original manuscript[1]. Because patients were retrospectively stratified and many German transplant centers do not routinely perform liver biopsy in thalassemia patients before transplant, information on liver fibrosis was limited to some patients. In addition, information on chelation therapy was limited in a few patients from foreign countries. Therefore, the patients were assigned into six risk categories: class 1, class 1-2, class 2, class 2-3, class 3, and patients ≥ 16 years of age. Liver iron concentration (LIC) before HSCT was measured by biomagnetic liver susceptometry or different magnetic resonance imaging methods. To allow its comparison, LIC results were converted into values (in mg/g dry weight) according to the Ferriscan® method.[2,3] Busulfan was always given intravenously, but analysis of busulfan-based protocols were limited due to missing information on therapeutic drug monitoring (area under the curve). Chimerism analysis was performed on unselected cells, usually in whole blood once a week up to day +100. Mean ciclosporin A (CSA) trough target level is the initially aimed level before adaptions were made due to GVHD, mixed chimerism or beginning of regular tapering.

Statistical methods

For analysis of overall survival (OS) failure was defined as death from any cause and surviving patients were censored at the date of last contact. The study had an explorative study design and most *P*-values are considered descriptive.

SUPPLEMENTARY RESULTS

Patient and donor characteristics

All 9/10 MMUD corresponded to a 7/8 HLA match. Last serum ferritin before HSCT was in the range of 102-7000 µg/l (median: 1800 µg/l) and LIC[2,3] before HSCT in the range of 1.2-21.3 mg/g dry weight (median 5.8 mg/g dry weight).

Transplant characteristics

Pre-conditioning therapy in 53 patients consisted of either hydroxyurea (n=7), azathioprine (n=2), hydroxyurea-azathioprine (n=41) or dexamethasone-fludarabine (+/-hydroxyurea) (n=3). The indication for pre-conditioning varied among transplant centers. Several centers have administered pre-conditioning not adjusted to the Pesaro risk class. Of 53 patients who received pre-conditioning about half belonged to low-risk patients (Pesaro Risk class 1 or 2 with Ferritin below 3000µg/l and age below 16 years). Four different regimens of busulfan-fludarabine-based protocols were applied namely busulfan-fludarabine (n=7), busulfan-fludarabine-thiotepa (n=18), busulfan-fludarabine-cyclophosphamide (n=2), or busulfan-fludarabine-cyclophosphamide-thiotepa (n=5). The treosulfan-fludarabine-thiotepa conditioning protocol was applied in 92 patients and relatively homogenous including treosulfan (36-)42g/m^2, fludarabine 150-160 (-180) mg/m^2 and thiotepa 8-10 mg/m2. Ex-vivo T-cell depletion of the graft consisted in one patient of CD3CD19 negative selection and in nine patients of CD34 positive selection with CD3+ T-Cell add back. One patient received a TCRalpha/beta depleted graft. Normal applications of MTX as GvHD prophylaxis comprised three applications in the vast majority of patients, seldom four. Omitted MTX applications were replaced by a course of MMF in the majority of patients. One patient with a MMUD received a concept with post-transplantation cyclophopsphamide (busulfan, fludarabine, ATG, PTCY, CSA and MMF). Large differences existed between initially aimed, CSA trough target

levels between centers. Of those centers that performed MSD- as well as UD-HSCTs, only one center had chosen different (slightly higher) CSA trough target levels for UD-HSCTs. The median duration of initial therapy with ciclosporin A after HSCT was 133 days, and that of any calcineurin-inhibitor was 156 days.

Graft failure and chimerism

Of eight patients that suffered from graft failure with renewed transfusion-dependency, seven patients received second HSCT and one patient entered chronic transfusion program with chelation therapy. Of seven patients with second HSCT, four patients are alive without graft failure and without chronic GvHD, one patient died after second HSCT suffering from severe acute and chronic GvHD, and two patients are alive but rejected the graft. One received successful third HSCT, the other entered chronic transfusion program with chelation therapy. Seven of eight patients with graft failure had a last donor chimerism (before 2nd HSCT) of <10%. One patient had a last donor chimerism of 12% three months before 2nd HSCT. The median follow up of the lowest chimerism analysis in 58 patients with mixed chimerism occurred at 88 days after HSCT. The median follow up of the last chimerism analysis was performed 735 days after HSCT. In five of nine patients who received boost+/-DLI, the main reason for cell therapy was bone marrow insufficiency and not a mixed chimerism. Whereas, 14 patients received DLI or boost +/-DLI due to a mixed donor percentage. Of these 14, four suffered from graft failure. In 2/14 patients the percentage of donor chimerism dropped further but stabilized at 15-20%. In five patients donor chimerism stabilized and increased slightly (between 6-20%). Three patients converted to complete chimerism after DLI/boost application. Of these 14 patients, 10 had a MSD (2xGF; 2 x dropping and stabilized; 4 x stable; 2 x conversion to complete chimerism).

Graft-versus-host disease (GvHD)

Ten patients received donor lymphocyte infusions (without boost) due to mixed chimerism. None was followed by graft-versus-host disease. An allogeneic boost +/- donor lymphocyte infusion was given to nine patients. One patient with busulfan-fludarabine-based conditioning suffered from acute GvHD grade II° starting four weeks thereafter.

Complications

Two patients, both with high dose of ATG, pre-conditioning and a MMUD, developed post-transplant lymphoproliferative disease and were successfully treated with rituximab. One patient with MSD-HSCT, high dose ATG and pre-conditioning suffered from Ewing sarcoma 29 months after HSCT.

Comparison of BF-based and TFT conditioning concepts

Of thirteen patients who received busulfan-fludarabine or busulfan-fludarabine-thiotepa without pre-conditioning (and relatively low CD3+ cell count in the graft (median 3.8 x $10E^7$/kg)) none experienced MC below 75%.

REFERENCES

1. Lucarelli G, Galimberti M, Polchi P, Angelucci E, Baronciani D, Giardini C, et al. Bone marrow transplantation in patients with thalassemia. N Engl J Med. 1990;322:417-421.
2. Fischer R, Piga A, Harmatz P, Nielsen P. Monitoring long-term efficacy of iron chelation treatment with biomagnetic liver susceptometry. Ann N Y Acad Sci. 2005;1054:350-357.

3. St Pierre TG, Clark PR, Chua-anusorn W, Fleming AJ, Jeffrey GP, Olynyk JK, et al. Noninvasive measurement and imaging of liver iron concentrations using proton magnetic resonance. Blood. 2005;105:855-861.

Supplementary Table 1. Additional patient and HSCT characteristics as well as outcome in the entire cohort of 124 patients and differences according to donor subgroup.

	**n (%) Total N=124**	**n (%) MSD N=57**	**n (%) MFD N=10**	**n (%) MUD N=27**	**n (%) MMUD N=30**	**Descrip-tive *P***
LIC according to Ferriscan, mg/g dry weight, n (%)						
≤ 5.0	36 (43.4)	17 (42.5)	2 (33.3)	6 (42.9)	11 (47.8)	0.758
5.1-10.0	20 (24.1)	7 (17.5)	1 (16.7)	4 (28.6)	8 (34.8)	
10.1-15.0	18 (21.7)	10 (25.0)	2 (33.3)	3 (21.4)	3 (13.0)	
>15.0	9 (10.8)	6 (15.0)	1 (16.7)	1 (7.1)	1 (4.3)	
Not reported	41	17	4	13	7	
R/D sex match, n (%)						
Male/male	40 (32.3)	16 (28.1)	2 (20.0)	12 (44.4)	10 (33.3)	0.256
Female/female	34 (27.4)	14 (24.6)	4 (40.0)	8 (29.6)	8 (26.7)	
Female/male	22 (17.7)	9 (15.8)	1 (10.0)	3 (11.1)	9 (30.0)	
Male/female	28 (22.6)	18 (31.6)	3 (30.0)	4 (14.8)	3 (10.0)	
R/D CMV status, n (%)						
Neg /neg	20 (16.1)	8 (14.0)	0 (0.0)	6 (22.2)	6 (20.0)	0.259
Neg /pos	15 (12.1)	4 (7.0)	3 (30.0)	4 (14.8)	4 (13.3)	
Pos / pos	75 (60.5)	38 (66.7)	7 (70.0)	12 (44.4)	18 (60.0)	
Pos /neg	14 (11.3)	7 (12.3)	0 (0.0)	5 (18.5)	2 (6.7)	
R/D EBV status, n (%)						
Neg /neg	6 (6.8)	6 (14.3)	0 (0.0)	0 (0.0)	0 (0.0)	0.027
Neg /pos	26 (29.6)	6 (14.3)	3 (37.5)	10 (62.4)	7 (31.8)	
Pos / pos	44 (50.0)	22 (52.4)	5 (62.5)	5 (31.3)	12 (54.6)	
Pos /neg	12 (13.6)	8 (19.0)	0 (0.0)	1 (6.3)	3 (13.6)	
Not reported	36	15	2	11	8	
Use of ATG, n (%)						
Yes	119 (96.0)	52 (91.2)	10 (100.0)	27 (100.0)	30 (100.0)	0.153
No	5 (4.0)	5 (8.8)	0 (0.0)	0 (0.0)	0 (0.0)	
Dosage of ATG administration, n (%)						
High	77 (64.7)	34 (65.3)	4 (40.0)	19 (70.4)	20 (66.7)	0.413
Low	42 (35.3)	18 (34.6)	6 (60.0)	8 (29.6)	10 (33.3)	
MNC, $x10^8$/kg, median (range)						
BM only	3.7 (1.1-10.1)	2.9 (1.2-10.1)	4.8 (2.4-8.9)	4.5 (1.8-9.6)	4.0 (1.1-7.7)	<0.001
PBSC with TCD	2.6 (0.1-28.7)	-	3.8	2.5 (0.3-11.7)	2.6 (0.1-28.7)	
PBSC without TCD	8.0 (3.4-22.1)	3.5 (3.4-5.0)	-	15.6 (7.9-22.1)	8.3 (6.2-20.9)	
All except CB	3.8 (0.1-28.7)	3.2 (1.2-10.1)	4.7 (2.4-8.9)	4.7 (0.3-22.1)	5.6 (0.1-28.7)	0.004
CD34+, $x10^6$/kg, median (range)						
BM only	5.1 (0.6-19.0)	5.2 (1.4-19.0)	3.2 (1.4-8.3)	4.9 (0.6-14.5)	5.1 (1.8-13.0)	0.009
PBSC with TCD	9.4 (4.5-16.2)	-	4.5	9.3 (6.4-16.2)	9.7 (4.9-15.8)	
PBSC without TCD	7.0 (3.6-23.0)	5.4 (5.4-7.0)	-	9.6 (9.0-23.0)	6.2 (3.5-19.1)	
All except CB	5.4 (0.6-23.0)	5.2 (1.4-19.0)	3.8 (1.4-8.3)	5.8 (0.6-23.0)	5.6 (1.8-19.1)	0.076

	n (%) Total N=124	n (%) MSD N=57	n (%) MFD N=10	n (%) MUD N=27	n (%) MMUD N=30	Descrip-tive *P*
Median follow up, years	3.2	3.0	3.0	3.1	5.0	0.899
Median time to ANC>0.5/nl, days, (range)	22 (9-52)	25 (10-46)	20 (16-34)	22 (11-52)	20 (9-31)	0.009
Median time to platelet count >20/nl, days, (range)	27 (12->100)	32 (16->100)	24 (17->100)	27 (12-144)	23 (13->100)	0.829
CMV reactivation, n/N, only in patients at higher risk (1y-probability, %)	27/89 (30.3)	9/45 (20.0)	2/7 (28.6)	6/17 (35.3)	10/20 (51.8)	0.037
EBV reactivation, n/N, only in patients at higher risk (1y-probability, %)	6/70 (8.6)	0/28 (0.0)	0/8 (0.0)	2/15 (13.3)	4/19 (21.1)	0.033
SOS, n (%)	8 (6.5)	6 (10.5)	1 (10.0)	0 (0.0)	1 (3.3)	0.205
TRM-1st HSCT, n (%)	4 (3.2)	0 (0)	0 (0)	1 (3.7)	3 (10)	0.077
Cause of death						
Infection without GvHD	2	0	0	0	2	
GvHD (+ infection)	2	0	0	1	1	
GvHD at 2nd HSCT after GF at 1st HSCT	1	0	0	0	1	

ATG antithymocyte globulin, *ANC* absolute neutrophil count, *BM* bone marrow, *CB* cord blood, *CMV* cytomegalovirus, *D* donor, *EBV* Epstein Barr virus, *HSCT* hematopoietic stem cell transplantation, *LIC* liver iron concentration, *MFD* matched family donor other than sibling, *MNC* mononuclear cells, *MSD* matched sibling donor, *MUD* matched unrelated donor 10/10, *MMUD* mismatched unrelated donor 9/10, *PBSC* peripheral blood stem cells, *R* recipient, *SOS* hepatic sinusoidal obstruction syndrome, *TCD* ex vivo T-cell depletion, *TFS* thalassemia-free survival, *TGFS* thalassemia-free survival without persisting chronic GvHD at last follow up, *TRM* treatment related mortality.

Supplementary Table 2. Univariable analysis of overall survival, thalassemia-free survival, graft failure, lowest donor chimerism and graft-versus-host disease analyzed in the entire cohort (N=124).

**Outcome**<br>***Overall survival***	***4y-OS, % (95% CI)***	**Descriptive *P***<br>***Log-rank***
Age at HSCT (years)		
<12	97.8 (85.3-99.7)	0.012
≥12	89.7 (74.9-96.0)	
Donor		
MSD	100.0 (NA)	0.029
MFD	100.0 (NA)	
MUD	96.3 (76.5-99.5)	
MMUD	84.7 (63.3-94.2)	
Pre-Conditioning		
Yes	92.5 (81.1-97.1)	0.072
No	97.4 (82.8-99.6)	
GvHD prophylaxis		
CSA only	100.0 (NA)	0.062
CSA + standard MTX dosage	98.5 (89.6-99.8)	
CSA + MTX with unknown dosage	100.0 (NA)	
CSA + reduced MTX (+/-MMF)	77.8 (36.5-93.9)	
CNI + MMF	100.0 (NA)	
Three agents	87.1 (54.4-96.9)	
Thalassemia-free survival	***4y-TFS, % (95% CI)***	***Log-rank***
Sex		
Male	98.5 (89.6-99.8)	0.001
Female	80.4 (67.3-88.6)	
Donor		
MSD	96.5 (86.7-99.1)	0.082
MFD	90.0 (47.3-98.5)	
MUD	88.9 (69.4-96.3)	
MMUD	79.9 (60.5-90.4)	
Graft failure	***4y-Probability GF, % (95% CI)***	***Log-rank***
Sex		
Male	1.5 (0.2-10.4)	0.010
Female	13.1 (6.5-25.5)	
Conditioning		
BF-based	0.0 (NA)	0.090
TFT	8.9 (4.6-17.0)	
Pre-Conditioning		
Yes	0.0 (NA)	0.015
No	11.3 (5.8-21.3)	
Stem cell source		
PBSC unmanipulated	0.0 (NA)	0.017
Cord blood (+/- bone marrow)	0.0 (NA)	
Bone marrow	5.5 (2.3-12.6)	
PBSC with TCD	27.3 (9.7-62.9)	
Mean targeted trough level of CSA (µg/l)		
≤100 (low)	0.0 (NA)	0.075
101-149 (medium)	4.2 (1.4-12.6)	
≥150 (high)	15.0 (6.5-32.4)	

Acute GvHD III-IV	*1y-Probability aGvHD III-IV, % (95% CI)*	*Log-rank*
Mean targeted trough level of CSA (µg/l)		
≤100	28.6 (11.8-59.4)	0.068
101-149	8.3 (3.8-17.6)	
≥150	8.8 (2.9-24.9)	
Donor		
MSD	0.0 (NA)	0.003
MFD	10.0 (1.5-52.7)	
MUD	22.2 (10.7-42.9)	
MMUD	20.7 (9.9-40.4)	
GvHD prophylaxis		
CSA only	0.0 (NA)	0.066
CSA + normal MTX	4.7 (1.5-13.8)	
CSA + red. MTX (+/-MMF)	33.3 (12.2-71.8)	
CSA + MTX (amount unclear)	0.0 (NA)	
CNI + MMF	13.6 (4.6-36.6)	
Three agents	16.7 (6.6-38.5)	
Extensive chronic GvHD	***4y-Probability extCGvHD, % (95% CI)***	***Log-rank***
Serum ferritin before HSCT, µg/l		
<3000	7.2 (3.2-15.8)	0.022
≥3000	21.1 (9.3-43.4)	
Acute GvHD III-IV		
No	7.1 (3.4-14.7)	0.001
Yes	35.7 (14.9-70.2)	

Only variables with a *P* of <0.1 are depicted.
aGvHD acute graft versus host disease, *BF-based* busulfan-fludarabine-based, *CI* confidence interval, *CNI* Calcineurin Inhibitor, *CSA* ciclosporin A, *extCGvHD* extended chronic graft versus host disease, *GF* graft failure, *HSCT* hematopoietic stem cell transplantation, *MFD* matched family donor other than sibling, *MMF* mycophenolate mofetil, *MSD* matched sibling donor, *MUD* matched unrelated donor 10/10, *MMUD* mismatched unrelated donor 9/10, *MWU* Mann Whitney U Test, *NA* not applicable, *OS* overall survival, *PBSC* peripheral blood stem cells, *red MTX* reduced methotrexate, *TCD* ex vivo T-cell depletion, *TFS* thalassemia-free survival, *TFT* treosulfan, fludarabine and thiotepa.

Supplementary Table 3. Univariable analysis of graft failure analyzed in patients with TFT conditioning (N=92).

**Outcome**<br>***Graft failure***	***4y-Probability GF, % (95% CI)***	**Descriptive *P***<br>***Log-rank***
Sex		
Male	2.1 (0.3-13.9)	0.012
Female	17.0 (8.5-32.5)	
Pre-Conditioning		
Yes	0.0 (NA)	0.030
No	13.8 (7.2-25.8)	
Stem cell source		
PBSC unmanipulated	0.0 (NA)	0.009
Cord blood (+/- bone marrow)	0.0 (NA)	
Bone marrow	7.6 (3.2-17.3)	
PBSC with TCD	37.5 (13.9-77.1)	
Mean targeted trough level of CSA (µg/l)		
≤100	0.0 (NA)	<0.001
101-149	4.9 (1.6-14.4)	
≥150	41.7 (19.9-73.0)	
CD3+ in the graft, x 10^7/kg		
≥6.0	0.0 (NA)	0.074
<6.0	13.7 (6.4-27.9)	

Only variables with a *P* of <0.1 are depicted.
CI confidence interval, *CSA* ciclosporin A, *GF* graft failure, *NA* not applicable, *PBSC* peripheral blood stem cells, *TCD* ex vivo T-cell depletion, *TFT* treosulfan, fludarabine and thiotepa.

Supplementary Table 4. Differences in patient and HSCT characteristics as well as outcome according to donor subgroups in 92 patients with TFT conditioning.

	**n (%) MSD N=40**	**n (%) MFD N=7**	**n (%) MUD N=22**	**n (%) MMUD N=23**	**Descrip-tive *P***
Patient age at HSCT, years, median (range)	8.1 (2.1-28.1)	6.2 (1.4-12.3)	7.7 (1.7-23.7)	9.8 (2.0-18.1)	0.445
Patient sex, female, n (%)	16 (40.0)	3 (42.9)	10 (45.5)	14 (60.9)	0.457
Serum ferritin ≥ 3000 µg/l, n(%)	6 (15.0)	1 (14.3)	2 (9.1)	5 (22.7)	0.686
LIC according to Ferriscan, mg/g dry weight, n (%)					
≤ 5.0	15 (53.6)	2 (50.0)	6 (50.0)	9 (52.9)	0.615
5.1-10.0	5 (17.9)	1 (25.0)	3 (25.0)	7 (41.2)	
10.1-15.0	5 (17.9)	0 (0.0)	2 (16.7)	1 (5.9)	
>15.0	3 (10.7)	1 (25.0)	1 (8.3)	0 (0.0)	
Not reported	12	3	10	6	
Pesaro class, n (%)					
1	2 (5.1)	0 (0.0)	1 (4.5)	0 (0.0)	0.354
1 or 2	16 (41.0)	5 (71.4)	14 (63.7)	17 (77.3)	
2	11 (28.2)	2 (28.6)	3 (13.6)	1 (4.5)	
2 or 3	5 (12.8)	0 (0.0)	2 (9.1)	1 (4.5)	
3	0 (0.0)	0 (0.0)	0 (0.0)	0 (0.0)	
≥ 16 years	5 (12.8)	0 (0.0)	2 (9.1)	3 (13.6)	
Not evaluable	1	0	0	1	
R/D sex match, n (%)					
Male/male	9 (22.5)	1 (14.3)	9 (40.9)	8 (34.9)	0.093
Female/female	10 (25.0)	2 (28.6)	7 (31.9)	7 (30.4)	
Female/male	6 (15.0)	1 (14.2)	3 (13.6)	7 (30.4)	
Male/female	15 (37.5)	3 (42.9)	3 (13.6)	1 (4.3)	
R/D CMV status, n (%)					
Neg /neg	7 (17.5)	0 (0.0)	4 (18.2)	4 (17.4)	0.592
Neg /pos	3 (7.5)	3 (42.9)	3 (13.6)	3 (13.0)	
Pos / pos	25 (62.5)	4 (57.1)	11 (50.0)	14 (60.9)	
Pos /neg	5 (12.5)	0 (0.0)	4 (18.2)	2 (8.7)	
R/D EBV status, n (%)					
Neg /neg	6 (20.0)	0 (0.0)	0 (0.0)	0 (0.0)	0.081
Neg /pos	5 (16.7)	2 (28.6)	9 (60.0)	7 (38.9)	
Pos / pos	16 (53.3)	5 (71.4)	5 (33.3)	9 (50.0)	
Pos /neg	3 (10.0)	0 (0.0)	1 (6.7)	2 (11.1)	
Not reported	10	0	7	5	
Stem cell source, n (%)					
BM only	34 (85.0)	7 (100.0)	16 (72.7)	11 (47.8)	0.003
Cord blood +/- BM	3 (7.5)	0 (0.0)	0 (0.0)	0 (0.0)	
PBSC without TCD	3 (7.5)	0 (0.0)	4 (18.2)	6 (26.1)	
PBSC with TCD	0 (0.0)	0 (0.0)	2 (9.1)	6 (26.1)	
MNC, $x10^8$/kg, median (range)					
BM only	3.4 (1.2-10.1)	4.8 (2.4-8.9)	4.8 (1.9-9.6)	5.4 (2.5-7.7)	<0.001
PBSC with TCD	-	-	7.1 (2.5-11.7)	2.2 (0.1-28.7)	
PBSC without TCD	3.5 (3.4-5.0)	-	15.6 (7.9-22.1)	8.6 (6.2-20.9)	
All except CB	3.4 (1.2-10.1)	4.8 (2.4-8.9)	5.0 (1.9-22.1)	5.9 (0.1-28.7)	0.004

	n (%) MSD N=40	n (%) MFD N=7	n (%) MUD N=22	n (%) MMUD N=23	Descrip-tive *P*
CD34+, x10^6/kg, median (range)					
BM only	4.5 (1.4-16.1)	5.4 (1.4-8.3)	5.0 (0.6-14.5)	5.1 (2.6-13.0)	0.028
PBSC with TCD	-	-	11.3 (6.4-16.2)	9.5 (4.9-15.8)	
PBSC without TCD	5.4 (5.6-7.0)	-	9.6 (9.0-23.0)	6.0 (3.6-19.1)	
All except CB	5.1 (1.4-16.1)	5.4 (1.4-8.3)	6.1 (0.6-23.0)	5.8 (2.6-19.1)	0.093
CD 3+, x10^7/kg, median (range)					
BM only	3.9 (1.5-9.3)	5.4 (1.4-7.4)	4.5 (2.6-30.4)	5.3 (2.1-13.3)	<0.001
PBSC with TCD	-	-	5.0 (5.0-5.1)	3.0 (0.1-5.5)	
PBSC without TCD	15.0	-	41.8 (20.0-52.5)	26.8 (18.2-50.6)	
All except CB	4.0 (1.5-15.0)	5.4 (1.4-7.4)	5.0 (2.6-52.5)	5.5 (0.1-50.6)	0.095
Pre-conditioning					
Yes	16 (40.0)	1 (14.3)	9 (40.9)	8 (34.8)	0.639
No	24 (60.0)	6 (85.7)	13 (59.1)	15 (65.2)	
Conditioning					
Treo 42g/m^2	35 (87.5)	4 (57.1)	22 (100.0)	20 (87.0)	0.025
Treo 36g/m^2	5 (12.5)	3 (42.9)	0 (0.0)	3 (13.0)	
Use of ATG, n (%)					
Yes	35 (87.5)	7 (100.0)	22 (100.0)	23 (100.0)	0.149
No	5 (12.5)	0 (0.0)	0 (0.0)	0 (0.0)	
Dosage of ATG administration, n (%)					
High	20 (57.1)	1 (14.3)	14 (63.6)	16 (69.6)	0.079
Low	15 (42.9)	6 (85.7)	8 (36.4)	7 (30.4)	
GvHD prophylaxis, n (%)					
CSA only	3 (7.5)	0 (0.0)	0 (0.0)	0 (0.0)	0.772
CSA + normal MTX	26 (65.0)	5 (71.4)	18 (81.8)	14 (61.0)	
CSA + red MTX +/- MMF	3 (7.5)	1 (14.3)	2 (9.1)	3 (13.0)	
CSA + MTX (amount unclear)	0 (0.0)	0 (0.0)	0 (0.0)	0 (0.0)	
CNI + MMF	6 (15.0)	1 (14.3)	2 (9.1)	3 (13.0)	
Three agents (CSA + MMF + red MTX/Steroid	2 (5.0)	0 (0.0)	0 (0.0)	3 (13.0)	
Mean targeted trough level of CSA, µg/l, n (%)					
≤100 (low)	8 (21.6)	0 (0.0)	4 (18.2)	2 (8.7)	0.279
101-149 (medium)	27 (73.0)	6 (85.7)	15 (68.2)	15 (65.2)	
≥150 (high)	2 (5.4)	1 (14.3)	3 (13.6)	6 (26.1)	

.

	n (%)<br>MSD<br>N=40	n (%)<br>MFD<br>N=7	n (%)<br>MUD<br>N=22	n (%)<br>MMUD<br>N=23	Descrip-tive *P*
Median follow up in years	2.7	4.1	3.1	5.0	0.856
Median time to ANC>0.5/nl, days, (range)	25 (10-40)	25 (20-34)	23 (11-52)	20 (9-31)	0.006
Median time to platelet count >20/nl, days, (range)	29 (16-54)	25 (17-34)	27 (15-144)	23 (13->100)	0.477
Graft failure, n (4y-probability, %)	2 (5.0)	1 (14.3)	2 (9.1)	3 (13.9)	0.625
Lowest chimerism, n (%)					
95-100%	13 (32.5)	3 (42.9)	16 (72.8)	18 (81.8)	0.001
75-94%	14 (35.0)	0 (0.0)	2 (9.1)	0 (0.0)	
50-74%	3 (7.5)	1 (14.2)	1 (4.5)	0 (0.0)	
<50%	10 (25.0)	3 (42.9)	3 (13.6)	4 (18.2)	
Not evaluable	0	0	0	1	
Last chimerism, n (%)					
95-100%	24 (60.0)	3 (42.9)	17 (77.3)	18 (81.8)	0.049
75-94%	8 (20.0)	0 (0.0)	2 (9.1)	0 (0.0)	
50-74%	2 (5.0)	2 (28.6)	0 (0.0)	0 (0.0)	
<50%	6 (15.0)	2 (28.6)	3 (13.6)	4 (18.2)	
Not evaluable	0	0	0	1	
Cell therapy, n (%)					
None	31 (77.5)	6 (85.7)	18 (81.8)	21 (95.5)	0.153
Autologous rescue	0 (0.0)	0 (0.0)	0 (0.0)	1 (4.5)	
Only DLI	6 (15.0)	0 (0.0)	1 (4.5)	0 (0.0)	
Allogeneic Boost +/- DLI	3 (7.5)	1 (14.3)	3 (13.6)	0 (0.0)	
Not evaluable	0	0	0	1	
aGvHD, n (1y-probability, %)					
II-IV					
III-IV	2 (5.0)	1 (14.3)	4 (18.2)	9 (40.9)	0.002
	0 (0.0)	1 (14.3)	4 (18.2)	5 (22.7)	0.024
extCGvHD, n (4y-probability, %)	2 (7.6)	1 (14.3)	2 (10.1)	2 (9.5)	0.822
CMV reactivation, n/N, only in patients at higher risk (1y-probability, %)	5/30<br>(16.7)	1/4<br>(25.0)	5/15<br>(33.3)	6/16<br>(39.4)	0.306
EBV reactivation, n/N, only in patients at higher risk, (1y-probability, %)	0/21<br>(0.0)	0/7<br>(0.0)	1/14<br>(7.1)	4/16<br>(27.3)	0.023
SOS, n (%)	3 (7.5)	1 (14.3)	0 (0.0)	0 (0.0)	0.187
Complications*, n (%)	8 (20.0)	1 (14.3)	8 (36.4)	10 (43.5)	0.161
TRM-1st HSCT, n (%)	0 (0.0)	0 (0.0)	1 (4.5)	2 (8.7)	0.226
4-year overall survival, %	100.0	100.0	95.5	84.8	0.128
Cause of death					
Infection without GvHD	0	0	0	1	
GvHD (+ infection)	0	0	1	1	
GvHD at 2nd HSCT after GF at 1st HSCT	0	0	0	1	
4-year TFS, %	95.0	85.7	86.4	78.3	0.230
4-year TGFS, %	92.5	85.7	86.4	73.9	0.198

*including aGvHD III-IV, cGvHD, mechanical ventilation, oxygen therapy, inotropic support, dialysis, SOS, encephalopathy, ileus, gastrointestinal bleeding.

ATG antithymocyte globulin, *aGvHD* acute graft versus host disease, *ANC* absolute neutrophil count, *BM* bone marrow, *CB* cord blood, *CNI* Calcineurin Inhibitor, *CMV* cytomegalovirus, *CSA* ciclosporin A, *D*

donor, *DLI* donor lymphocyte infusion, *EBV* Epstein Barr virus, *extCGvHD* extended chronic graft versus host disease, *GvHD* graft versus host disease, *HSCT* hematopoietic stem cell transplantation, *LIC* liver iron concentration, *MFD* matched family donor other than sibling, *MMF* mycophenolate mofetil, *MNC* mononuclear cells, *MSD* matched sibling donor, *MUD* matched unrelated donor 10/10, *MMUD* mismatched unrelated donor 9/10, *PBSC* peripheral blood stem cells, *R* recipient, *red MTX* reduced methotrexate, *SOS* hepatic sinusoidal obstruction syndrome, *TCD* ex vivo T-cell depletion, *TFS* thalassemia-free survival, *TFT* treosulfan, fludarabine and thiotepa, *TGFS* thalassemia-free survival without persisting chronic GvHD at last follow up, *TRM* treatment related mortality.

Supplementary Table 5. Risk factors of CMV reactivation in 89 patients at higher risk analyzed by multivariable cox regression analysis.

	Events/evaluable	Hazard ratio (95% CI)	*P*
Donor			
MSD	9/45	1.0	
MFD	2/7	1.60 (0.34-7.56)	0.551
MUD	6/17	1.56 (0.48-5.04)	0.454
MMUD	10/20	5.02 (1.79-14.07)	0.002
ATG dosage			
Low	4/27	1.0	
High	22/57	3.68 (1.22-11.10)	0.021
Pre-Conditioning			
No	10/51	1.0	
Yes	17/38	3.34 (1.43-7.80)	0.005
aGvHD II-IV			
No	21/78	1.0	
Yes	6/10	1.88 (0.67-5.27)	0.231

All first-time CMV reactivations occurred within the first two months after HSCT. Only variables with $P<0.1$ in the univariable analysis were included in multivariable analysis.

aGvHD acute graft versus host disease, *ATG* antithymocyte globulin, *CI* confidence interval, *CMV* cytomegalovirus, *MFD* matched family donor other than sibling, *MSD* matched sibling donor, *MUD* matched unrelated donor 10/10, *MMUD* mismatched unrelated donor 9/10.

Supplementary Table 6. Additional differences in patient and HSCT characteristics as well as outcome in 124 patients receiving different conditioning regimens.

	n (%)<br>TFT<br>N=92	n (%)<br>BF-based<br>N=32	Descriptive *P*
LIC according to Ferriscan, mg/g dry weight, n (%)			
≤ 5.0	32 (52.5)	4 (18.2)	0.002
5.1-10.0	16 (26.2)	4 (18.2)	
10.1-15.0	8 (13.1)	10 (45.4)	
>15.0	5 (8.2)	4 (18.2)	
Not reported	31	9	
R/D sex match, n (%)			
Male/male	27 (29.3)	13 (40.6)	0.735
Female/female	26 (28.3)	8 (25.0)	
Female/male	17 (18.5)	5 (15.6)	
Male/female	22 (23.9)	6 (18.8)	
R/D CMV status, n (%)			
Neg/neg	15 (16.3)	5 (15.6)	0.949
Neg/pos	12 (13.0)	3 (9.4)	
Pos/pos	54 (58.7)	21 (65.6)	
Pos/neg	11 (12.0)	3 (9.4)	
R/D EBV status, n (%)			
Neg/neg	6 (8.6)	0 (0.0)	0.041
Neg/pos	23 (32.9)	3 (16.7)	
Pos/pos	35 (50.0)	9 (50.0)	
Pos/neg	6 (8.6)	6 (33.3)	
Not reported	22	14	
MNC, $x10^8$/kg, median (range)			
BM only	3.9 (1.2-10.1)	3.0 (1.1-5.6)	<0.001
PBSC with TCD	2.5 (0.1-28.7)	3.8 (0.3-6.9)	
PBSC without TCD	7.9 (3.4-22.1)	8.3 (8.0-8.6)	
All except CB	4.5 (0.1-28.7)	3.2 (0.3-8.6)	0.020
CD34+, $x10^6$/kg, median (range)			
BM only	5.1 (0.6-16.1)	5.1 (1.6-19.0)	0.006
PBSC with TCD	9.5 (4.9-16.2)	9.3 (4.5-11.4)	
PBSC without TCD	7.0 (3.6-23.0)	6.6 (5.6-7.7)	
All except CB	5.4 (0.6-23.0)	5.2 (1.6-19.0)	0.615
Use of ATG, n (%)			
Yes	87 (94.6)	32 (100.0)	0.326
No	5 (5.4)	0 (0.0)	
Dosage of ATG administration, n (%)			
High	51 (58.6)	26 (81.3)	0.030
Low	36 (41.4)	6 (18.7)	
Median follow up in years	3.2	3.2	0.382
Median time to ANC >0.5/nl, days, (range)	22.0 (9-52)	19.0 (13-46)	0.576
Median time to platelet count >20/nl, days, (range)	25.0 (15->100)	33.0 (12->100)	0.006
CMV reactivation, n/N, only in patients at higher risk, (1y-probability, %)	17/65 (26.5)	10/24 (41.7)	0.153
EBV reactivation, n/N, only in patients at higher risk, (1y-probability, %)	5/58 (8.8)	1/12 (9.1)	0.944
SOS, n (%)	4 (4.3)	4 (12.5)	0.203

	n (%)<br>TFT<br>N=92	n (%)<br>BF-based<br>N=32	Descriptive *P*
TRM-1st HSCT, n (%)	3 (3.3)	1 (3.1)	1.0
Cause of death			
Infection without GvHD	1	1	
GvHD (+ infection)	2	0	
GvHD at 2nd HSCT after GF at 1st HSCT	1	0	

ATG antithymocyte globulin, *ANC* absolute neutrophil count, *BF-based* busulfan-fludarabine-based, *BM* bone marrow, *CB* cord blood, *CMV* cytomegalovirus, *D* donor, *EBV* Epstein Barr virus, *HSCT* hematopoietic stem cell transplantation, *LIC* liver iron concentration, *MMF* mycophenolate mofetil, *MNC* mononuclear cells, *PBSC* peripheral blood stem cells, *R* recipient, *SOS* hepatic sinusoidal obstruction syndrome, *TCD* ex vivo T-cell depletion, *TFT* treosulfan, fludarabine and thiotepa, *TRM* treatment related mortality.

a

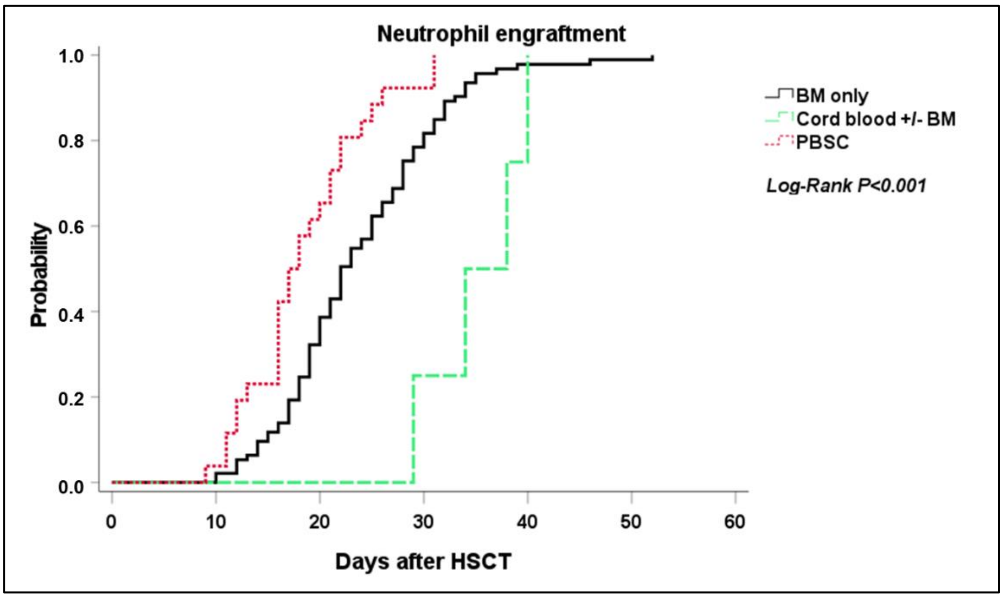

b

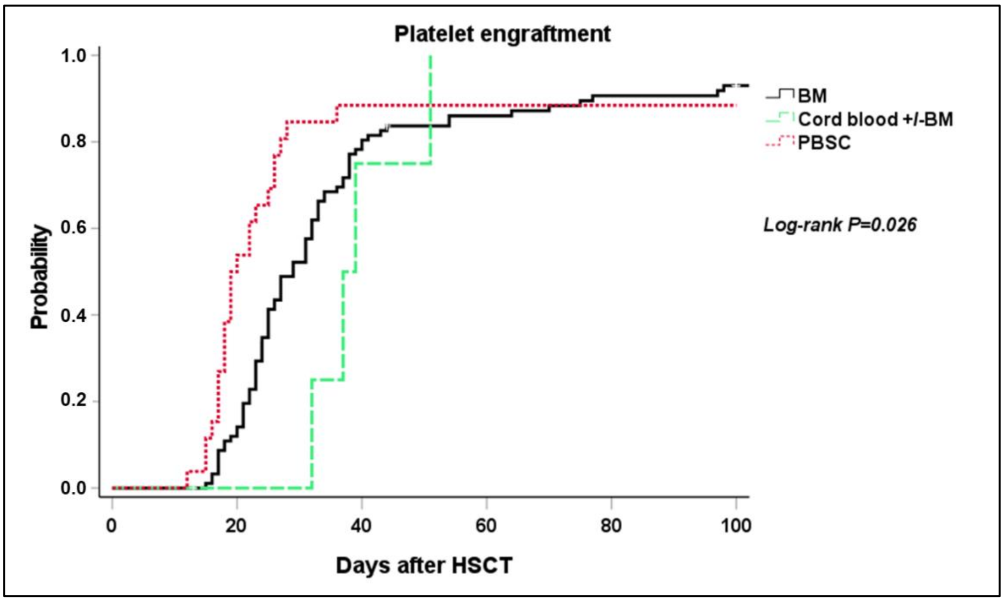

Supplementary Fig. 1. Neutrophil and platelet engraftment. (a) Neutrophil engraftment and (b) platelet engraftment according to graft source in the entire cohort.

a

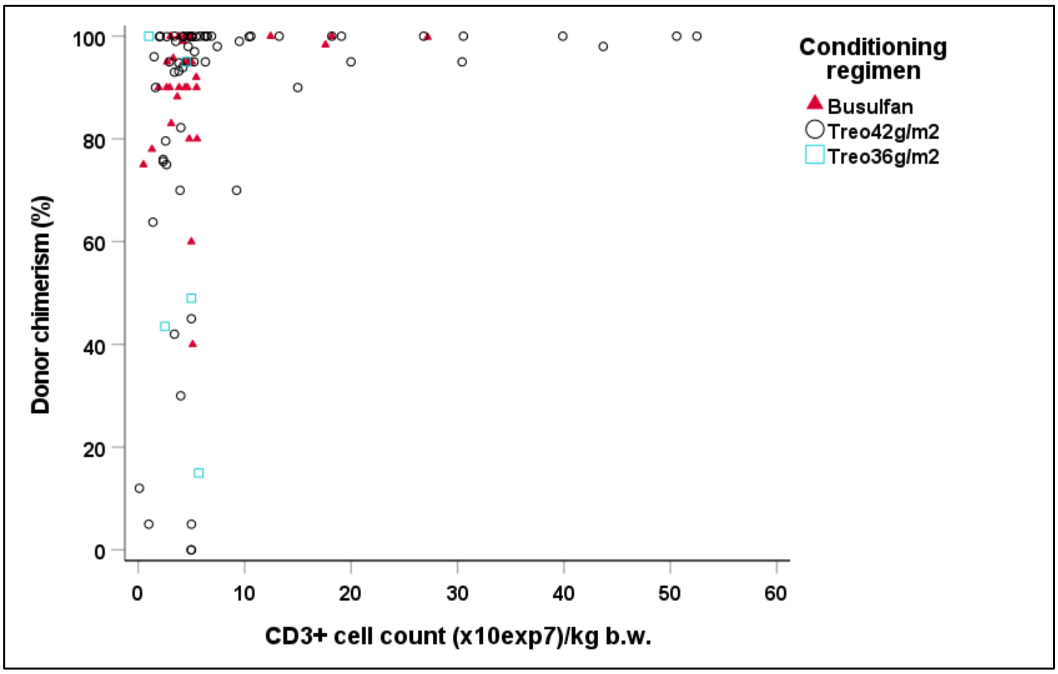

b

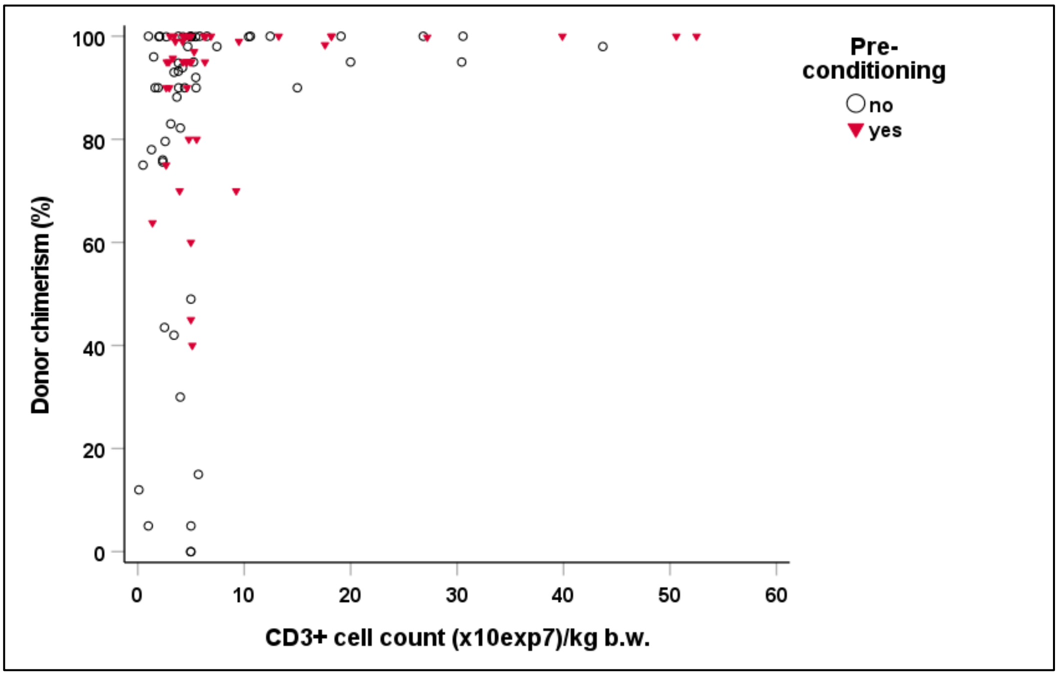

Supplementary Fig. 2. Relationship of CD3+ cell count (x10^7/kg body weight) in the graft and percentage of lowest donor chimerism. 99 patients with available CD3+ cell count are depicted with the specification of (a) conditioning regimen and (b) pre-conditioning.
